# Transformation-Induced Creep and Creep Recovery of Shape Memory Alloy

**DOI:** 10.3390/ma5050909

**Published:** 2012-05-22

**Authors:** Kohei Takeda, Hisaaki Tobushi, Elzbieta A. Pieczyska

**Affiliations:** 1 Department of Mechanical Engineering, Aichi Institute of Technology, 1247 Yachigusa, Yakusa-cho, Toyota 470-0392, Japan; E-Mail: tobushi@aitech.ac.jp; 2 Institute of Fundamental Technological Research, Polish Academy of Sciences, Pawinskiego 5B, Warsaw 02-106, Poland; E-Mail: epiecz@ippt.gov.pl

**Keywords:** shape memory alloy, superelasticity, subloop, transformation band, creep, creep recovery, local deformation

## Abstract

If the shape memory alloy is subjected to the subloop loading under the stress-controlled condition, creep and creep recovery can appear based on the martensitic transformation. In the design of shape memory alloy elements, these deformation properties are important since the deflection of shape memory alloy elements can change under constant stress. The conditions for the progress of the martensitic transformation are discussed based on the kinetics of the martensitic transformation for the shape memory alloy. During loading under constant stress rate, temperature increases due to the stress-induced martensitic transformation. If stress is held constant during the martensitic transformation stage in the loading process, temperature decreases and the condition for the progress of the martensitic transformation is satisfied, resulting in the transformation-induced creep deformation. If stress is held constant during the reverse transformation stage in the unloading process, creep recovery appears due to the reverse transformation. The details for these thermomechanical properties are investigated experimentally for TiNi shape memory alloy, which is most widely used in practical applications. The volume fraction of the martensitic phase increases in proportion to an increase in creep strain.

## 1. Introduction

Shape memory alloys (SMAs) are remarkable materials characterized by the thermomechanical properties of shape memory and superelasticity. Since properties like these are highly conducive to the functions of smart materials, their applications have attracted worldwide attention [1,2,3]. However, in order for SMAs to be applied effectively in the design of shape memory elements, the thermomechanical properties of the material have to be taken into account. The functional properties of an SMA appear based on the martensitic transformation (MT), and since the MT is sensitive to variations in temperature and stress and to their hysteresis, the deformation properties due to the MT are complex [4,5]. Research up to now in this area has been mainly concerned with a full loop (or perfect loop) of the MT completion. But in practical applications, temperature and stress are likely to vary in various ranges. If SMA elements are subjected to loads with a subloop (or partial loop, internal loop) in which temperature or stress varies in an incomplete MT range, the conditions for the start and finish of the MT as in a full loop are not satisfied. That is to say, the conditions for the progress of the MT will therefore change depending on the previous hysteresis of temperature and stress [6,7,8,9]. An example of this would be a case in which an SMA element accomplishes a two-way movement depending on an MT of cooling and a reverse MT of heating, both under constant stress. In a full loop description, the SMA element undergoes an amount of deformation corresponding to the maximum stress-induced martensitic transformation (SIMT) strain. However, in the case of a subloop, the SMA element does not complete the whole of this stroke. Similarly, since the recovery stress which occurs in an SMA element subjected to heating and cooling under a constant strain will depend on the hysteresis of the temperature, the actual variation in the recovery stress for a given subloop will be smaller than that obtained in a full loop. It can be recognized from this that the subloop deformation behavior of an SMA is of great importance for an accurate evaluation of the functional properties of SMA elements and for the design of such elements for practical applications.

The present study investigates superelastic deformation behaviors of TiNi alloy, the most widely used SMA in practical applications, in various subloop loading tests, in particular the dependence of the subloop deformation on the loading rate, and the characteristics of transformation-induced creep and creep recovery deformation in the stress plateau region under constant stress. Variations in the SIMT bands during deformation are observed using a microscope, and a thermograph is used to identify the temperature distributions on the surface of the tape specimen. The subloop deformation behaviors are discussed in terms of the local deformations due to the SIMT.

## 2. Conditions for Progress of Martensitic Transformation

The transformation kinetics for the MT in SMAs proposed by Tanaka *et al*. is expressed as follows [4,10]

(1) $$\frac{\dot{z}}{1-z}=b_{M}C_{M}\dot{T}-b_{M}\dot{\sigma }\geq 0$$

and for the reverse transformation

(2) $$-\frac{\dot{z}}{z}=b_{A}C_{A}\dot{T}-b_{A}\dot{\sigma }\geq 0$$

where *σ*, *T* and *z* represent the stress, temperature and the volume fraction of the martensitic phase (M-phase), respectively. The volume fraction of the parent phase or austenitic phase (A-phase) is 1-*z*. An overdot denotes the time derivative. The material parameters *b_M_*, *C_M_*, *b_A_* and *C_A_* are determined from the experiments.

The conditions for start and finish of the MT are expressed by the following equations, respectively.

(3) $$\sigma =C_{M}\left(T-M_{s}\right)$$

(4) $$\sigma =C_{M}\left(T-M_{f}\right)$$

The conditions for the reverse transformation are as follows, respectively.

(5) $$\sigma =C_{A}\left(T-A_{s}\right)$$

(6) $$\sigma =C_{A}\left(T-A_{f}\right)$$

The parameters *M_s_*, *M_f_*, *A_s_* and *A_f_* denote the start and finish temperatures for the MT and the reverse transformation under no load, respectively.

From Equation (1), the condition for progress of the MT becomes as follows since *b_M_* < 0.

(7) $$\frac{d\sigma }{dT}>C_{M}\;:\;for\;dT>0\;\frac{d\sigma }{dT}<C_{M}\;:\;for\;dT<0$$

From Equation (2), the condition for progress of the reverse transformation becomes as follows since *b_A_* > 0.

(8) $$\frac{d\sigma }{dT}<C_{A}\;:\;for\;dT>0\;\frac{d\sigma }{dT}>C_{A}\;:\;for\;dT<0$$

**Figure 1:**
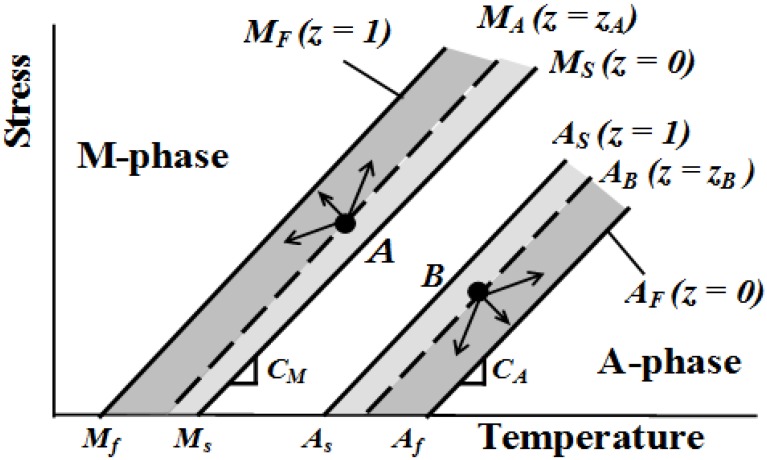
Conditions for progress of the martensitic transformation (MT) and the reverse transformation in the subloop loadings on the stress-temperature phase diagram.

The conditions for progress of the phase transformation in the subloop loading during the phase transformation are shown on the stress-temperature phase diagram in Figure 1. In Figure 1, *M_S_* (*z* = 0) and *M_F_* (*z* = 1) denote the MT start and finish lines with a slope of *C_M_*, respectively, and *A_S_* (*z* = 1) and *A_F_* (*z* = 0) denote the reverse transformation start and finish lines with a slope of *C_A_*, respectively. Points *A* and *B* in Figure 1, respectively, represent the state of progress of the MT and the reverse transformation and the volume fractions of the M-phase at each point are *z_A_* and *z_B_*. The broken lines *M_A_* and *A_B_* denote the states with the volume fractions *z_A_* and *z_B_*, respectively. The conditions described by Equations (7) and (8) for progress of the phase transformation from the points *A* and *B* mean that stress and temperature vary in the directions shown by the arrows in Figure 1.

## 3. Experimental Method

### 3.1. Materials and Specimens

The material used in the tests was a TiNi alloy containing Ti-50.95 at.% Ni. The specimens were in the form of a polycrystalline tape of this material produced by Furukawa Techno Material Co., Ltd. in Japan. The transformation temperatures *A_s_*, *A_f_*, *R_s_*, *R_f_*, *M_s_* of the material obtained from the DSC test were 254 K, 281 K, 281 K, 258 K, 220 K. The thickness and the width of the tape were uniform, at 0.7 mm and 10 mm, respectively. The final cold-rolling rate was 25% and the heat-treatment temperature was 803 K for 1 min. The specimens used in the test were of two gage lengths, 50 mm and 100 mm, where “gage length” (GL) means the distance between the two securing grips. The aspect ratios (the ratios of GL:width) of these two types of specimens were thus 5 and 10, respectively.

The surface of the specimens used to observe the SIMT bands was mirror-like, finished with a No. 4000 emery paper. The surface used to detect the temperature distributions by means of infrared thermography was covered with a thin black layer of carbon powder.

### 3.2. Experimental Apparatus

The testing apparatus for SMA characteristics appearing in the tension test consisted of a tension machine and a heating-cooling device in which heating and cooling can be achieved by hot air and cold air, respectively. A digital camera (DMX-HD2, Sanyo Co.) and a motion analysis microscope (VW-6000, Keyence Corporation) were used for the observation of SIMT bands on the surface of the specimen and an infrared thermography device (NEO Thermo TVS-700, Nippon Avionics Co., Ltd.) was used for the detection of heating or cooling effects due to exothermic or endothermic reactions to the SIMT. The strain measurement during tensile tests is calculated from stroke displacement.

### 3.3. Experimental Procedure

Three kinds of tension tests for subloop loading were carried out in air at room temperature (295–299 K). During the tests, the SIMT band characteristics and the temperature distributions on the surface of the specimens were measured continuously. The three kinds of tests were:

(1) Subloop loading test at different loading rates: With respect to the maximum strain of 8% at the upper stress plateau of the stress-strain curve, loading and unloading tests were conducted at constant rates of strain and stress and the subloop deformation behaviors were observed for different loading rates.
(2) Creep test: Stress loading was applied at a constant rate up to a level of 2% strain at the upper stress plateau, and stress was then kept constant while the creep deformation behavior was observed.
(3) Creep and creep recovery test: Stress loading was applied at a constant rate up to a strain of 2% at the upper stress plateau, and stress was then kept constant while the creep deformation was observed. After reaching the maximum strain due to the creep deformation, stress was removed at the constant rate down to a decrease in strain of 1.5% from the maximum strain at the lower stress plateau during unloading, and stress was then kept constant while the creep recovery behavior was observed.

## 4. Superelastic Deformation under Constant Strain Rate

**Figure 2:**
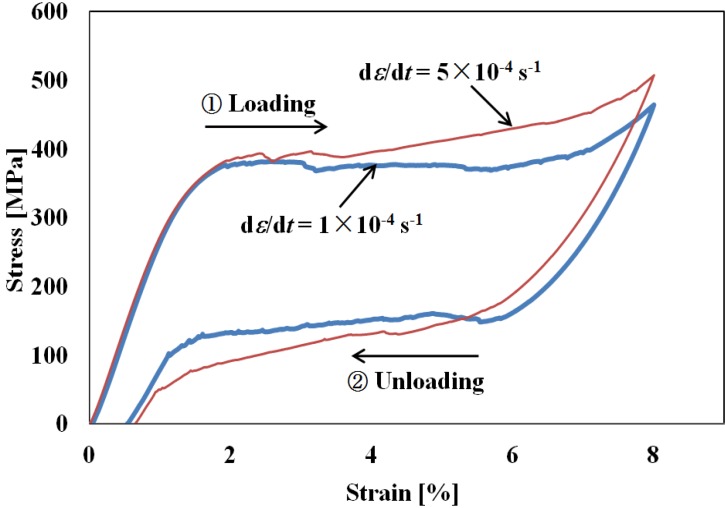
Stress-strain curves under strain rates of d*ε*/d*t* = 1 × 10^−4^ s^−1^ and d*ε*/d*t* = 5 × 10^−4^ s^−1^.

The stress-strain curves obtained from the tension tests under constant strain rates of 1 × 10^−4^ s^−1^ and 5 × 10^−4^ s^−1^ are shown in Figure 2, in which the stress-strain curves represent the hysteresis loops during loading and unloading. Strain is recovered in the unloading process, indicating superelasticity. At a low strain rate, an upper and a lower stress plateaus appear, due to the SIMT during loading in the upper case and to the reverse transformation during unloading in the lower one. At a high strain rate, the slope of the stress-strain curve in the stress plateau region is steeper, and the starting and finishing points of the SIMT and reverse transformation are less distinct. The increase in steepness at the higher strain rate can be explained in the case of the loading process by saying that there is an increase in temperature due to the SIMT which raises stress above the level needed, at the upper plateau, for the SIMT to progress in conditions of temperature constancy [11]. Similarly, in the unloading process at a high strain rate, the reverse transformation causes the stress to descend to a lower level than would otherwise occur at the lower stress plateau. This dependence of the slope of the stress-strain curve on the strain rate in the stress plateau regions also holds at strain rates higher than 5 × 10^−4^ s^−1^ [11,12,13,14].

## 5. Subloop Deformation under Constant Stress Rate

**Figure 3:**
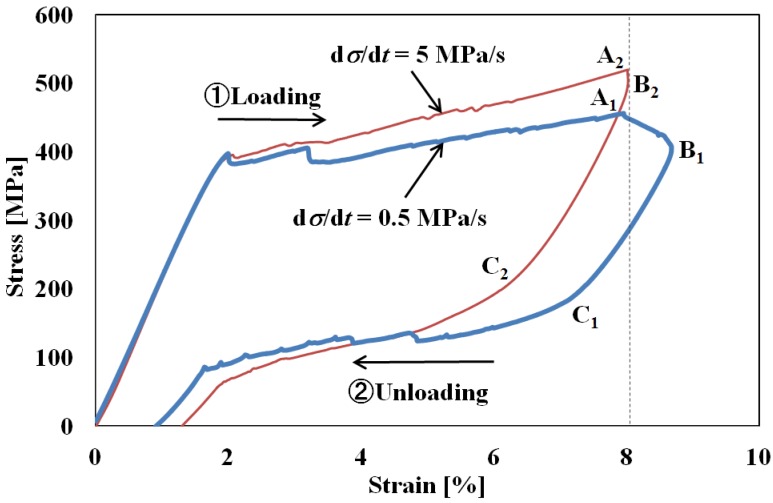
Stress-strain curves under stress rates of d*σ*/d*t* = 0.5 MPa/s and d*σ*/d*t* = 5 MPa/s.

**Figure 4:**
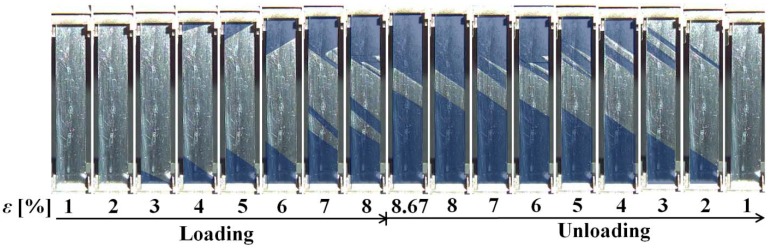
Photographs of specimen surface at various strains *ε* under stress rate of 0.5 MPa/s.

The stress-strain curves obtained from the tension tests under constant stress rates of 0.5 MPa/s and 5 MPa/s are shown in Figure 3. As before, these curves represent the hysteresis loops during loading and unloading and indicate superelasticity. Again, the slope in the stress plateau region is steeper for the higher stress rate. At the lower rate, from point A_1_ in the unloading process where the strain is 8%, strain initially increases to 8.67% at point B_1_ and then decreases to point C_1_, which is the starting point of the reverse transformation, as a result of elastic deformation. The initial increase in strain from A_1_ to B_1_ is due to the fact that the conditions for the SIMT to progress are still being satisfied in this early part of the unloading process as a carry-over from the rise in temperature produced by the SIMT in the loading process up to A_1_; as the unloading proceeds, the temperature then starts to come down [6,9]. At the higher stress rate, for which the rate of decrease in the stress is larger during the initial stage of unloading from point A_2_, any carried over increase in strain from the SIMT will be slight and balanced out by the decrease in elastic strain. As a result of these two counteracting variations in strain, stress decreases under almost constant strain down to point B_2_, after which strain decreases elastically down to point C_2_. Figure 4 shows photographs of specimen surfaces at various strains taken by a digital camera in the tension test under a stress rate of 0.5 MPa/s in the tension test. In Figure 4 the bands left by the SIMT (SIMT bands) have been enhanced with blue tinting. This is because the propagation patterns of these bands, although obvious enough to naked-eye observation, do not show up well in monochrome photographs. As can be observed from the series of images in Figure 4, SIMT bands, similar to Luders’ bands but at a certain angle of inclination, appear first at both ends and then spread toward the center. In the course of unloading from a controlled strain of 8%, the SIMT bands initially go on spreading up to a strain level of 8.67%, but then, as the unloading effect sets in, the center boundaries recede, causing the bands to shrink back toward both ends. Comparing the area fractions of the SIMT bands at the same strain levels in the loading and unloading phases, the transformed area is greater in unloading.

## 6. Transformation-Induced Creep

### 6.1. Creep Behavior

**Figure 5:**
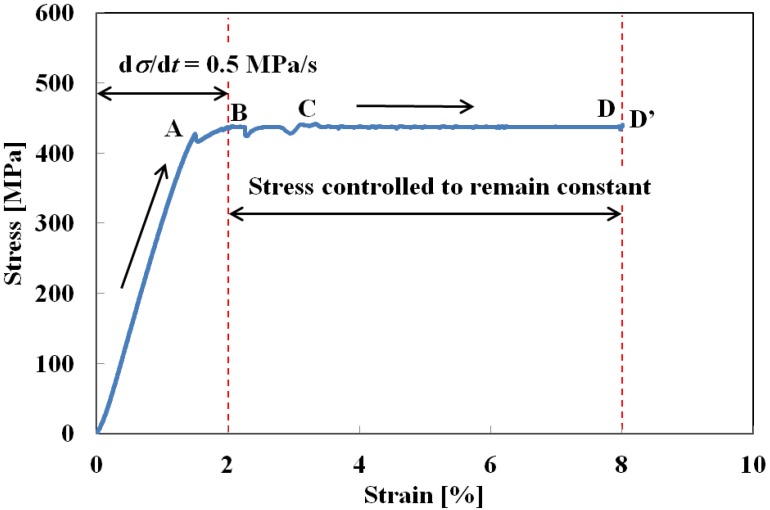
Stress-strain curve under stress rate of d*σ*/d*t* = 0.5 MPa/s till strain of 2% followed by stress controlled to remain constant.

Figure 5 shows the stress-strain curve obtained from the creep test under a constant stress rate of 0.5 MPa/s up to a strain of 2% at the upper stress plateau, followed by a constant stress. In Figure 5, the SIMT starts at a strain of 1.3% (point A) in the loading process, under a constant stress rate. If stress is controlled so as to remain constant at its level for 2% strain (point B), it initially fluctuates slightly before settling down to a constant 438 MPa at a strain of 3.5% (point C). Strain then continues to increase to about 8% (point D). This phenomenon of strain increase under constant stress is similar to what is found with normal creep deformation. The explanation in this case would be that the SIMT causes the temperature to increase during loading up to a strain of 2%, after which it decreases under a constant stress. Conditions are therefore satisfied for the SIMT to progress as discussed in Section 2 and strain increases.

**Figure 6:**
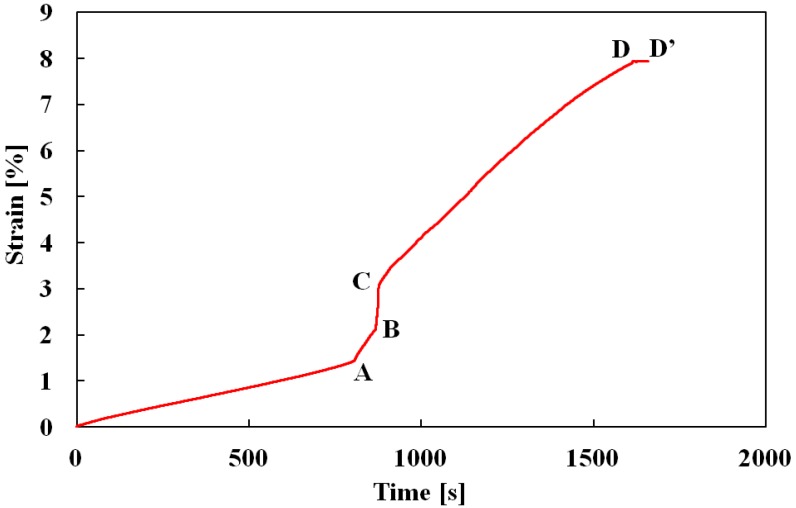
Variation in strain with passing of time in creep test.

The relationship between strain and time is shown in Figure 6. As can be seen in Figure 5 and Figure 6, the rate of increase in the strain rises sharply at the level of 1.3%, following the start of the SIMT. Stress fluctuates slightly between strain levels 2% and 3.5% while strain increases rapidly. After a strain of 3.5% is reached, stress settles down to be constant and strain increases at an almost constant rate of 6.5 × 10^−5^ s^−1^. Strain goes on increasing to about 8% before finally becoming constant (points D-D’). 

### 6.2. Progress of Creep

**Figure 7:**
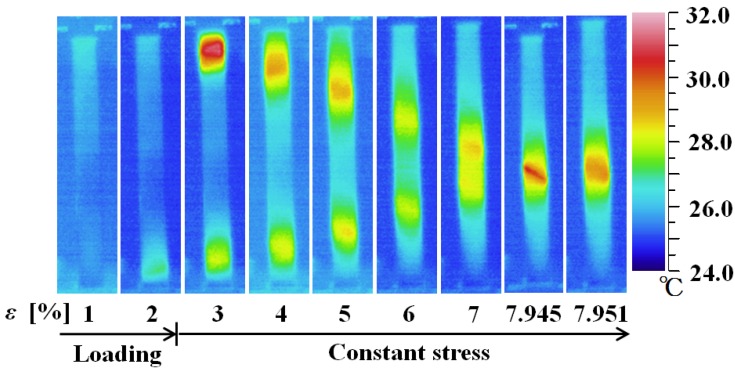
Thermograms of temperature distribution on the specimen surface under d*σ*/d*t* = 0.5 MPa/s up to a strain *ε* of 2% followed by constant stress.

**Figure 8:**
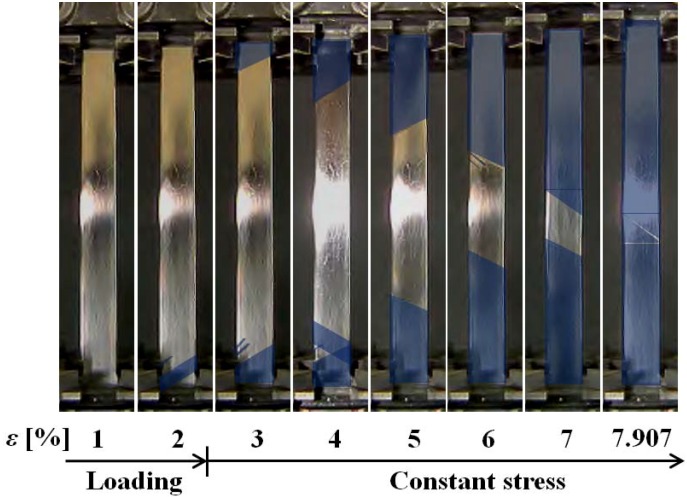
Photographs of specimen surface at various strains *ε* under stress rate of d*σ*/d*t* = 0.5 MPa/s up to a strain *ε* of 2% followed by constant stress.

Figure 7 shows thermograms of the temperature distributions on the surface of a specimen, and Figure 8 shows photographs of the SIMT bands.

As can be seen from the temperature distributions, the SIMT process due to the exothermic reaction first appears at the two ends during loading at a strain level of 2%, and then spreads toward the center where the bands combine into one, completing the SIMT. When the stress is held constant at the level reached for 2% strain, the SIMT bands spread due to a decrease in temperature. Transformation heat is generated at each new point of advance in the SIMT process, which leads to a chain reaction in the SIMT, resulting in creep deformation.

**Figure 9:**
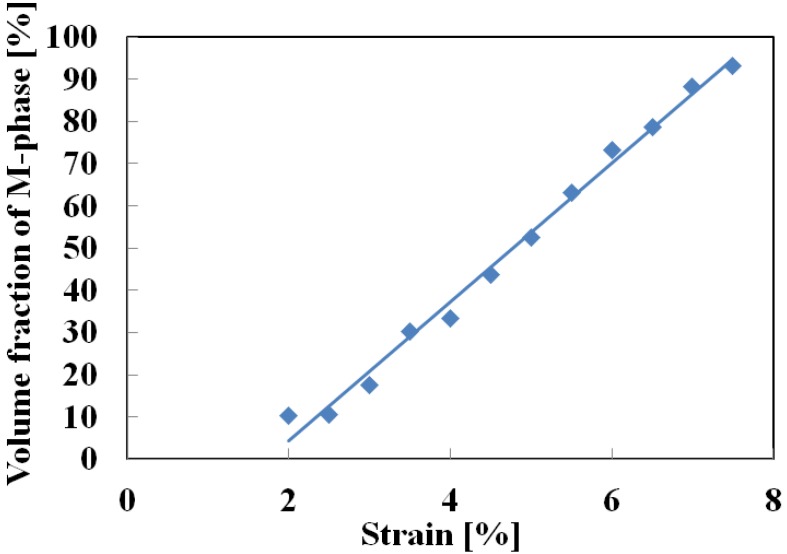
Relationship between volume fraction of M-phase and strain during creep deformation.

In Figure 8, the SIMT bands in the photographs taken by a motion analysis microscope are again tinted to enhance the visibility of the propagation progress. After first appearing at the two ends, the bands spread toward the center as stress is held constant. Once the whole surface has been transformed to the M-phase at a strain of about 8%, the strain stops growing. The inclined angle of the SIMT bands in the central part of the specimen is 42° which is close to the 45° suggested by Huang [15]. As can be seen from the previous Figure 5, when the SIMT bands combine in the center and strain stops increasing (point D), stress decreases by 5 MPa. All of the SIMT bands photographed in Figure 8 also appear in the same positions on the reverse surface of the specimen and can be considered as continuing throughout the cross section of the tape. This means that the area fraction occupied by the M-phase on each surface must be equivalent to the volume fraction occupied in the body as a whole. In this way, the volume fraction of the M-phase can be estimated from the measured area fraction of the SIMT bands. The relationship between the volume fraction of the M-phase and strain is shown in Figure 9. The volume fraction of the M-phase increases in proportion to an increase in strain.

## 7. Transformation-Induced Creep and Creep Recovery

**Figure 10:**
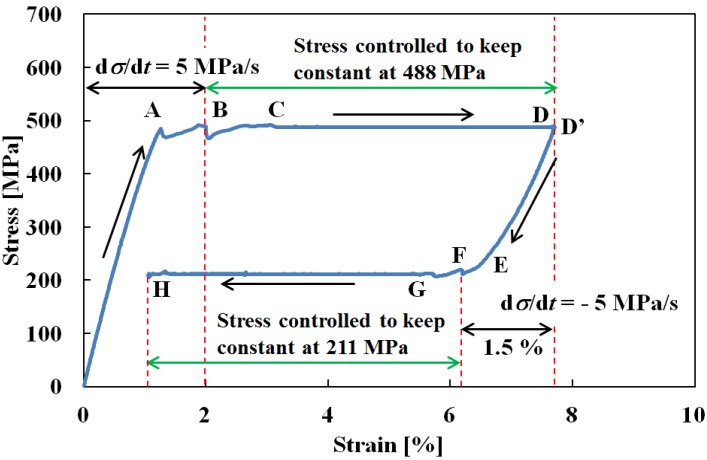
Stress-strain curve under stress rate of d*σ*/d*t* = 5 MPa followed by stress keeping from a strain of 2% during loading and stress keeping from decrease in strain of 1.5% from the maximum strain during unloading.

Figure 10 shows the stress-strain curve obtained from the creep and creep recovery test in which stress loading was applied under a constant stress rate of 5 MPa/s up to a strain of 2% at the upper stress plateau, followed by a constant stress till a stop of creep strain, and then stress was removed under a constant stress rate of −5 MPa/s down to a decrease in strain of 1.5% from the maximum strain at the lower stress plateau during unloading, followed by a constant stress till a stop of creep recovery. In Figure 10, the SIMT starts at point A and stress is controlled to keep constant at point B. Stress settles down to be constant at point C and strain increases to point D. This strain behavior under constant stress is similar to what was observed for creep deformation in Figure 5. Strain stops to increase at the point D and keeps constant with passing of time to point D’. In the unloading process, strain decreases elastically from point D’ to point E at which the reverse transformation starts. If stress is controlled so as to remain constant at its level for a decrease in strain of 1.5% from the maximum strain (point F), it initially fluctuates slightly before settling down to a constant 211 MPa at a strain of 5.8% (point G). Strain then continues to decrease to about 1% (point H). This phenomenon of strain decrease under constant stress is similar to what is found with normal creep recovery deformation. The explanation in this case would be that the reverse transformation causes the temperature to decrease during unloading down to point F, after which it increases under a constant stress. Conditions are therefore satisfied for the reverse transformation to progress as discussed in Section 2, and strain decreases.

**Figure 11:**
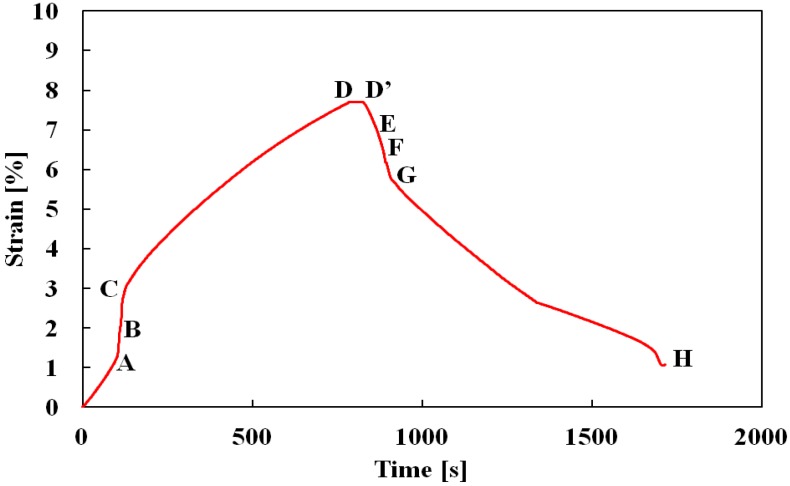
Variation in strain with passing of time in the creep and creep recovery test.

The relationship between strain and time is shown in Figure 11. As can be seen in Figure 10 and Figure 11, the strain from point A to point D with passing of time in the loading process is similar as observed in Figure 5 and Figure 6. Average creep strain rate from point C to point D is 7.3 × 10 ^−5^ s^−1^. Strain becomes constant at point D followed by a constant value till point D’. In the unloading process from point D’, the rate of decrease in the strain rises sharply following the start of the reverse transformation at point E. Stress fluctuates slightly between point F and point G while strain decreases rapidly. After point G is reached, stress settles down to be constant and strain decreases gradually. Average creep recovery strain rate from point G to point H is −5.8 × 10 ^−5^ s^−1^. The creep and creep recovery strain rates depend on stress rate, strain and stress level kept constant during loading and unloading. Details of creep and creep recovery strain behavior are subjects for future research.

## 8. Conclusions

This paper reports investigations of various subloop behaviors including creep and creep recovery associated with superelastic deformation of a TiNi SMA tape based on evidence of local temperature variation as measured by thermography and on surface observations of SIMT bands made with a motion analysis microscope during tension tests. The results obtained can be summarized as follows.

(1) Upper and lower stress plateaus appear in the stress-strain curves during loading and unloading in association with the spreading and shrinking of SIMT bands on the surface of the tape specimens. In the case of unloading from the upper stress plateau under a low stress rate, an increase in strain occurs due to the spreading of the SIMT bands at the start of the unloading.
(2) If stress is held constant at the upper stress plateau after loading up to a strain of 2% under a constant stress rate, creep deformation occurs due to the spread of the SIMT process. The creep strain rate under constant stress is almost constant. It increases in proportion to the increase in the volume fraction of the M-phase.
(3) If stress is held constant at the lower stress plateau after unloading down to a decrease in strain of 1.5% from the maximum strain under a constant stress rate, creep recovery deformation occurs due to reverse transformation.

## References

[1] Funakubo H. (1987). Shape Memory Alloys.

[2] Otsuka K., Wayman C.M. (1998). Shape Memory Materials.

[3] Cismasiu C. (2010). Shape Memory Alloys.

[4] Tanaka K., Kobayashi S., Sato Y. (1986). Thermomechanics of transformation pseudoelasticity and shape memory effect in alloys. Inter. J. Plast..

[5] Raniecki B., Lexcellent C., Tanaka K. (1992). Thermodynamic models of pseudoelastic behaviour of shape memory alloys. Arch. Mech..

[6] Tanaka K., Nishimura F., Tobushi H. (1994). Phenomenological analysis on subloop in shape memory alloys due to incomplete transformations. J. Intell. Mater. Syst. Struct..

[7] Lin P.H., Tobushi H., Tanaka K., Hattori T., Makita M. (1994). Pseudoelastic behaviour of TiNi shape memory alloy subjected to strain variations. J. Intell. Mater. Syst. Struct..

[8] Tanaka K., Nishimura F., Hayashi T., Tobushi H., Lexcellent C. (1995). Phenomenological analysis on subloops and cyclic behavior in shape memory alloys under mechanical and/or thermal loads. Mech. Mater..

[9] Pieczyska E.A., Tobushi H., Nowacki W.K., Gadaj S.P., Sakuragi T. (2007). Subloop deformation behavior of tini shape memory alloy subjected to stress-controlled loadings. Mater. Trans..

[10] Tanaka K. (1986). A thermomechanical sketch of shape memory effect: One-dimensional tensile behavior. Res. Mechanica..

[11] Pieczyska E.A., Tobushi H., Gadaj S.P., Nowacki W.K. (2006). Superelastic deformation behaviors based on phase transformation bands in TiNi shape memory alloy. Mater. Trans..

[12] He Y.J., Yin H., Zhou R.H., Sun Q.P. (2010). Ambient effect on damping peak of NiTi shape memory alloy. Mater. Lett..

[13] Shaw J.A., Kyriakides S. (1995). Thermomechanical aspects of NiTi. J. Mech. Phys. Solids.

[14] He Y.J., Sun Q.P. (2010). Rate-dependent domain spacing in a stretched NiTi strip. Inter. J. Solids Struct..

[15] Huang W.M. (2005). Transformation front in shape memory alloys. Mater. Sci. Eng..

